# The Impact of Business Cycle on Health Financing: Subsidized, Voluntary and Out-of-Pocket Health Spending

**DOI:** 10.3390/ijerph17061928

**Published:** 2020-03-16

**Authors:** Hao Dong, Zhenghui Li, Pierre Failler

**Affiliations:** 1 School of Economics and Statistics, Guangzhou University, Guangzhou 510006, China; donghao@e.gzhu.edu.cn; 2 Guangzhou International Institute of Finance and Guangzhou University, Guangzhou 510006, China; 3 Economics and Finance Group, Portsmouth Business School, University of Portsmouth, Portsmouth PO1 3DE, UK; pierre.failler@port.ac.uk

**Keywords:** business cycle, health financing, subsidized, voluntary and out-of-pocket health spending, hierarchical linear model, structural equation modeling

## Abstract

Diverse types of healthcare systems in countries offer opportunities to explore the heterogeneous sources of health financing. This paper widely explores the effect of the business cycle on subsidized, voluntary and out-of-pocket health spending in 34 countries with different types of healthcare systems, by the methodology of hierarchical linear modeling (HLM). We use a panel of annual data during the years from 2000 to 2016. It further examines the business cycle-health financing mechanism by inquiring into the mediating effect of external conditions and innovative health financing, based on the structural equation modeling (SEM). The empirical results reveal that the business cycle harms subsidized spending, whereas its effect on voluntary and protective health spending is positive. Results related to the SEM indicate that the mediating effect of external conditions on the relationship between the business cycle and health financing is negative. However, we find that the business cycle plays a positive effect on health financing through innovative health financing channels. Thus, designing and implementing efforts to shift innovative health financing have substantial effects on the sustainability of healthcare systems.

## 1. Introduction

Concern about the impact of business cycle on global health financing is vital for achieving the sustainability of healthcare systems and Universal Health Coverage (UHC). According to the 2016 report of the World Health Organization (WHO), the primary objective of health financing is to achieve expected health outcomes or strengthen the health climate [1]. Business cycle theories have typically resorted exogenous shocks, such as income inequality, unemployment, economic uncertainty in order to generate such features of healthcare systems [2,3,4,5,6,7]. The existing literature has repeatedly found evidence of public or private health financing. However, we lack a framework to systematically analyze the impact of business cycle on global health financing.

This paper firstly evaluates the impact of the business cycle on different sources of health financing. Building on heterogeneities among different types of healthcare systems setup, we employ a hierarchical linear model (HLM) to snapshot the distributions of business cycle to health financing among different healthcare systems [8,9,10,11]. The healthcare system not only enables people of a country to achieve equity from using health services but also protects patients or households from “financial ruin” [12,13,14]. Equity in healthcare systems is heavily correlated to the primary goal of financial protection but is conceptually heterogeneous. On the one hand, the gap between the demand and actual use of health service elevates the inequity of healthcare systems [15]. Subsidized health spending could be regarded as a tool to get adequate access to health service and be called for strengthening the equity [16,17,18]. On the other hand, financial protection is at the center of efforts to enjoy healthcare service. Overall, financial protection should be distributed according to the financial incentive [19,20]. Voluntary health spending is a direct financial incentive for unnecessary medical treatment. Besides, it allows households to share the risk of medical costs [21,22,23]. Out-of-pocket health spending can be used to assess the extent of financial protection within a country [24]. Obviously, in the absence of information asymmetry and externality, the business cycle exerts a comparable effect on health financing among different types of healthcare systems. In this sense, an important question is, how the business cycle will be distributed to health financing among different healthcare systems.

This paper specializes a structural equation model (SEM) to show that external conditions and innovative health financing exert mediating effects towards business cycle-health financing mechanisms. External conditions are shaped by government action, public policy, and socioeconomic context. Theoretically, there are several factors that potentially affect health financing. On the one hand, the supply-side factors, such as government actions, human resource management and reception of patients, could help change the out-of-pocket health spending [19,25]. On the other hand, Sanger et al. [26] investigated the effect of economic burden on the formal payment for healthcare. Therefore, exploring intermediate effects of external condition could highlight the transferred channel between the business cycle and health financing. In terms of innovative health financing, Atun et al. [27] systematically investigated the innovative financing instruments for global health. Innovative health financing could be regarded as a tool to track down the national financing related to health categories. With the increasing reliance on innovative financing to sustain healthcare systems, the important question is what should be the way forward in improving the efficiency and number of innovative financing instruments used to augment health financing toward healthcare systems [28]. To date, we lack a deep understanding for the mediate effect of innovative financing, and such evidence is necessary to improve the share of innovative financing instruments in health spending. Thus, the aim of this study is to offer evidence to explore the effect of the business cycle on global health financing through external conditions and innovative financing channels among different countries.

To explore the patterns described by the business cycle-health financing mechanisms, this paper investigates the heterogeneity and channels on health financing in 34 countries over a 16-year period, 2000–2016. Accordingly, the high share of out-of-pocket health spending could better reflect a lack of financial risk protection for households, that is also continue to be a major source of health financing in countries. Taking into account heterogeneities among different types of healthcare systems, this paper divides sample countries into three groups by the mean value of the share of out-of-pocket health spending from 2000–2016 to measure the types of healthcare systems. Building on a mediating mechanisms setup, we also develop a framework based on the SEM to separately explore the channel role of external conditions and innovative health financing on the relationships between business cycle and health financing among different types of healthcare system within countries.

This study contributes to the healthcare system literature in some aspects. First, there is a need to comprehensively and comparatively explore the impact of business cycle on subsidized, voluntary and out-of-pocket health spending. Studies on global health financing comprehensively estimated the health spending disaggregated by sources and compared the expected and potential spending in healthcare systems [12]. Concerning the impartial and protective aspects, it is generally accepted that the influence on global health financing should not only assess the single source of health outcomes but also eliminate heterogeneity associated with economic status in countries. Thus, this paper widely explores the effect of the business cycle on subsidized, voluntary and out-of-pocket health spending in lower-middle, upper-middle, and high-income countries by the HLM. Furthermore, the present study is different from existing literature that it separately investigates the impact of the business cycle over global health financing through path variables. It could be regarded as an exploration for effective channels. These channels are used to capture the effect of the business cycle on health financing, or to infer if increased health financing is linked to more business cycle or vice versa. What is more, different business cycle-health spending mechanisms show a varying level of heterogeneous income countries.

**Figure 1:**
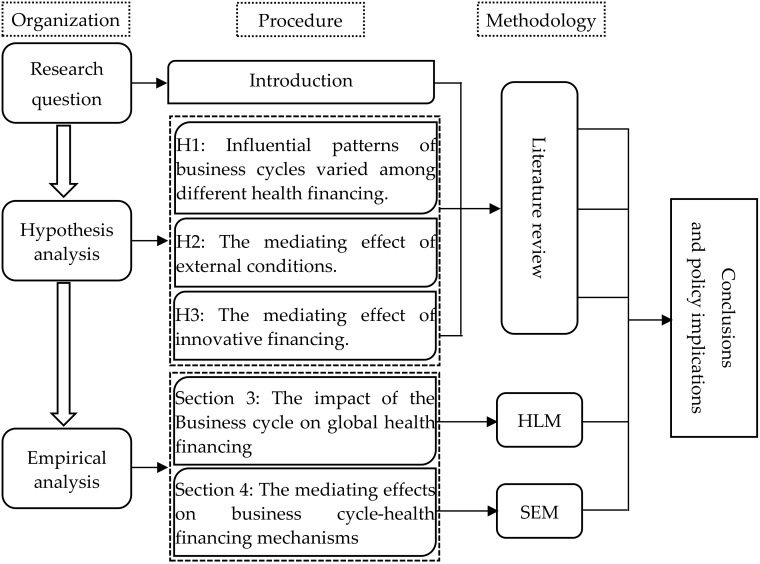
The logical organization of this paper.

The logical organization of the paper is shown in Figure 1. Section 2 presents the research hypothesis in this study. Section 3 depicts the impact of the business cycle on health financing in different countries. Furthermore, Section 4 analyzes the transfer channel between the business cycle and health financing. The conclusions and policy implications are shown in Section 5.

## 2. Research Hypothesis

**Figure 2:**
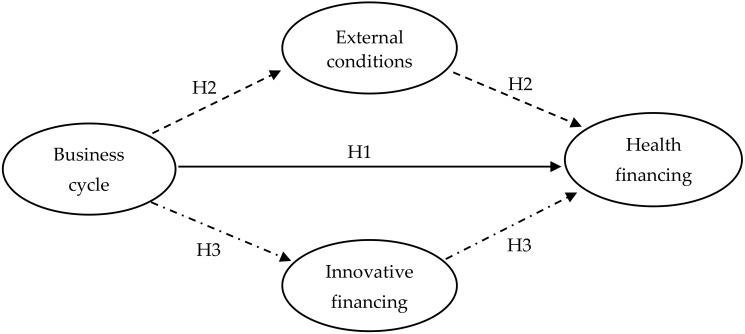
Hypothetical model.

Why have influential patterns of business cycles varied among different health financing? Figure 2 represents the logical organization of the hypothesis. In general, the business cycle affects health financing primarily through internal and external factors, such as broader issues of efficiency and market failure in healthcare systems, among different types of spending [29,30]. Specifically, the business cycle exerts negative effect on subsidized health spending. On the one hand, the negative effect related to the objective of subsidized spending. The primary aim of subsidized spending is to ensure the equality of consumers to assess the healthcare service [31,32]. To stabilize the healthcare system and improve its efficiency, governments or authorities began implementing ‘conversion period’ programs [33]. On the other hand, demands of health service also determine this negative effect. Consumers also could experience the change in their health quality according to the heterogeneity of working conditions during different phases of the business cycle [3,7,34]. The gaps existed in accessing to healthcare service elevates the negative effect of the business cycle on subsidized health spending [15].

The effect of the business cycle on voluntary and out-of-pocket health spending shows a different picture. These relationships between the business cycle and voluntary and out-of-pocket spending are determined by households’ after-tax-and-transfer income (ATTI) and health conditions. On the one hand, as the asymmetry of information, the increase of ATTI and the resulting uncertainty in economy may weaken the expectations of consumers and efficiency. This leads to a larger deterioration of voluntary and out-of-pocket health spending [35]. On the other hand, countries during different phases of the business cycle negatively influence their populations’ health conditions [36]. Less income effectively creates financial barriers to access health services, especially in healthcare systems heavily depend on out-of-pocket health spending [37]. Given the stability of the business cycle in countries with different types of healthcare systems, what is more, the expectation of participants are responsible for the negative effect of the business cycle on voluntary and out-of-pocket health spending [38].

Accordingly, this study presents the first hypothesis:

**Hypothesis** **1.** *Generally, the business cycle has a negative impact on subsidized spending, whereas the effect of the business cycle on voluntary and out-of-pocket health spending is positive*.

The question exists of how external conditions affect health financing could be grouped into two broad categories: the national fiscal position and consumers’ demand. From the perspective of the national fiscal position, this could include a surprise in fiscal risk or unforeseen financing constraints of healthcare systems [39]. On the one hand, external conditions could trigger cascades of events that heavily impact healthcare systems by changing unemployment and poverty, further resulting in the uncertainty of health spending [40,41,42]. On the other hand, external conditions may be regarded as unforeseen financing constraints of healthcare systems [43]. More specifically, external conditions exert uncertainty on working and living conditions, which could be seen as one of the barriers to access to healthcare service [3]. In terms of the consumers’ demand, consumers could expect to find higher wages to satisfy the normal and unexpected expenditure of healthcare service [6]. However, external conditions have also been related to higher prevalence of risk behaviors, such as smoking and less healthy lifestyles [44]. All these agreements highlight the determinant role of external conditions in health financing.

The relationships between business cycle and external conditions offer a unique opportunity to test the mediating effect of external conditions [45]. First, the relationship between the business cycle and the fiscal position of countries has witnessed this mediating effect. It is well-known that one country could be regarded as an expected regional economy if it timely and more professionally exposes the economic uncertainty in either the overall economy or the specific industry during different phases of the business cycle. This relationship has played an important role in reducing fiscal risks and financing constraints in healthcare systems [46]. Second, the consumers’ demand is heavily associated with the business cycle. A consumer’s demand for healthcare service responses to a shock could also be conceptualized as a diversified economy [30]. In this context, it would stand to reason that the business cycle has exposed the fundamental of economy, including fiscal position and the demand side of healthcare service, and eventually resulting in the changes in health financing.

As mentioned above, it could be clearly noted that:

**Hypothesis** **2.** *There is a mediating effect of external conditions on the relationship between the business cycle and health spending*.

Two priorities for meeting the health R&D highlight the crucial role of innovative financing in healthcare systems. Accordingly, two elements are recommended to explain the relationships between innovative health financing and health financing: a guarantee of sustainable financing and resource allocation [47]. On the one hand, innovative health financing offers a new opportunity for transferring traditional donor funding in novel ways to improve health financing [27]. Since innovative health financing enriches the ways that donors invest their funds, it is likely to increase in health spending. On the other hand, innovative health financing could be regarded as a shared global public resource [43]. Indeed, inequalities associated with regional and social resource allocation leave the poor with a greater unmet demand for healthcare services [48]. Another explanation is that innovative financing has effectively pooled and channeled financing from innovative health financing to beneficiary countries. For example, innovative instruments, such as exchange-traded funds failed to generate any valuable financing [49]. The Airline Levy, however, has become a steady source of global health financing. However, this situation is not applicable to rich countries, such as Norway, UK and USA. Consequently, there are heterogeneous effects of innovative health financing on health financing among different types of healthcare system.

Innovative health financing has been primarily channeled to relationships between the business cycle and health financing. Given the heterogeneous efficiency in countries with different types of healthcare systems, innovative financing will not be essential to stabilize the healthcare system, and it is difficult to collect international resources [50]. For one thing, economic uncertainty related to the business cycle will create risks for transferring traditional funds, which further lead to a major failure in the sustainability of health financing. For another, the business cycle also changes the conversion efficiency in healthcare systems as a result of the slowdown in the competitiveness of the economy and the shortage of resource allocation. With the commitment to the Millennium Development Goals, additionally, these disparities among different types of healthcare systems could intensify due to the heterogeneity of political instability, which is testing the effectiveness of healthcare systems in different countries [29]. In this vein, it could stand to reason that there are heterogeneous mediating effects on innovative health financing towards the impact of the business cycle on global health financing in countries with different types of healthcare systems.

Accordingly, this study concludes the last hypothesis:

**Hypothesis** **3.** *Innovative health financing mediates the association between the business cycle and global health financing*.

## 3. The Impact of the Business Cycle on Global Health Financing

### 3.1. Hierarchical Linear Model

A hierarchical linear model (HLM) can be employed as an effective tool to investigate the effect of the business cycle on global health financing among subsidized, voluntary and out-of-pocket health spending. Van and Nissen [51] developed a review of HLM in exploring the hierarchically nested structure in education datasets. Additionally, a large number of studies investigated the nested effect in the fields of environmental, firm and others [52,53]. However, sparse attention has been paid to the exploration of healthcare systems. Health financing is usually organized at a nested structure, namely, relationships between health financing and the business cycle are properly conceived as nests within the types of healthcare system. Actually, health financing and variables used in this paper (this could be introduced in Section 3.2) are tracking data that means they can be used as repeated measurements among multiple years in the countries. On the one hand, the characteristics of a country might be nested within the type of the healthcare system. On the other hand, these diverse types could, in turn, be nested within states. In other words, the business cycle could vary over different types of healthcare systems which are heavily related to the relationship between business cycle and external environment such as disease control and social stabilization. Moreover, a robust healthcare system is built to guarantee a fair access to health services and to prevent external shocks. Thus, this means we have to explore whether and how health financings are impacted by the interaction between the business cycle and types of the healthcare system. Using traditional linear models may ignore these nested characteristics which could lead to bias. Alternatively, the HLM can accomplish this by estimating unique models for each country with a different type of healthcare system. Thus, this paper uses the HLM. With the HLM, observation of different years forms the first level of HLM, whereas the type of healthcare system forms the second level. The specific models are as follows.

Level 1 could be expressed as (1):

(1) $$HSP_{t,i}=\pi_{0,i}+\pi_{1,i}BC_{t,i}+\pi_{2,i}UNF_{t,i}+\pi_{3,i}SER_{t,i}\;+\pi_{4,i}GOV_{t,i}+\pi_{5,i}UEM_{t,i}+\pi_{6,i}TEC_{t,i}+\pi_{7,i}SW_{t,i}+\varepsilon_{t,i}$$

Similarly, Level 2 could be shown as (2)-(3):

(2) $$\pi_{0,i}=\varphi_{00}+\varphi_{01}\times I_{H}+\varphi_{02}\times I_{M}+\tau_{t,i}$$

(3) $$\;\pi_{1,i}=\varphi_{10}+\varphi_{11}\times I_{H}+\varphi_{12}\times I_{M}\;$$

Mixed Model:

(4) $$HSP_{t,i}=\varphi_{00}+\varphi_{01}\times I_{H}+\varphi_{02}\times I_{M}\;+\varphi_{10}\times BC_{t,i}+\varphi_{11}\times I_{H}\times BC_{t,i}+\varphi_{12}\times I_{M}\times BC_{t,i}\;+\pi_{2,i}UNF_{t,i}+\pi_{3,i}SER_{t,i}++\pi_{4,i}GOV_{t,i}+\pi_{5,i}UEM_{t,i}+\pi_{6,i}TEC_{t,i}+\pi_{7,i}SW_{t,i}+\tau_{t,i}+\varepsilon_{t,i}$$

where $BC_{t,i}$ refers to the business cycle for country i at year t .$\;I_{H}$ and $I_{M}$ are dummies for countries with the high and medium share of out-of-pocket health spending. $UNF_{t,i}$ and $SER_{t,i}$ represent the public health conditions which are ‘Under Five Mortality Rate’ and ‘people using safely managed sanitation services’, respectively. $GOV_{t,i}\;$, $UEM_{t,i}$ and $TEC_{t,i}$ stand external conditions within a country which respectively are ‘government effectiveness’, ‘unemployment’ and ‘patent applications’. $SW_{t,i}$ represents structural weakness which is ‘compulsory financing arrangements’. Additionally, our interesting mainly lies in $\varphi_{10}\;$, $\varphi_{10}+\varphi_{11}$ and $\varphi_{10}+\varphi_{12}$ which respectively stand for the effect of the business cycle on health financing among different types of the healthcare system.

### 3.2. Data and Variables Selected

Accordingly, we use panel annual data of 34 countries from 2000 to 2016 because of the availability of data. We first depict the variables selected in this paper. Then, this paper presents the measurement of the healthcare system’s type. Specifically, a brief description of all variables used in this paper is shown in Table 1.

The dependent variables include subsidized, voluntary and out-of-pocket health spending. From the perspective of subsidized health spending, government health expenditure could be regarded as an effective tool to reduce the gaps between the individuals’ need and their ability to use the health service. In terms of voluntary health spending, voluntary financing arrangements depicts voluntary prepaid schemes to healthcare systems. Additionally, out-of-pocket expenditure indicates the individual’s ability-to-pay and refers to financial protection in a country. Specifically, the higher the share of out-of-pocket expenditure in total health expenditure, the more limited the financial protection in a country. In this sense, this paper selects government health expenditure of total general government expenditure, voluntary financing arrangements of current health expenditure and out-of-pocket expenditure of total expenditure obtained from Global Health Expenditure (GHE) database to measure the subsidized, voluntary and out-of-pocket health spending, respectively.

The main explanatory variable is the business cycle. Commonly, the gross domestic product is regarded as a measurement of business cycle, which can be obtained from IMF’s (International Monetary Fund) international financial statistic (IFS) database. Real GDP measurements are retrieved at constant prices and are used to calculate economic growth by removing the effect from general price levels within countries. Moreover, the Hodrick-Prescott filter (HP-filter) is a non-parametric method which is commonly used to filter trend and cycle components. To get a sense of the marginal effect of the business cycle on health financing, this paper employs HP-filtered real Gross Domestic Product (GDP) to compute the aggregate business cycle. Specifically, the business cycle was detrended from annual data using the HP-filter with a smoothing parameter of 100, which could be regarded as an effective parameter to snapshot the characteristics of GDP.

The incidence of health spending relates to different health conditions and socioeconomy. Arguably, the crucial factors of health financing are public health conditions, economic conditions and structural weakness [5,20,43]. In terms of public health, this paper uses the Under Five Mortality Rate (UNF) and People using safely managed sanitation services (SER) obtained from world development index (WDI) database, since they could capture perceptions of the health of people. At a specific level, the UNF concerns about the global monitoring of child mortality, and the SER depicts the percentage of people using improved sanitation facilities that are not shared with other households. As a proxy for the external conditions, this paper selects Government Effectiveness (GOV), Unemployment (UEM) and Patent Applications (TEC). The UEM, collected in the WDI database, is not only a signal of economic distress but plays a key role in increasing health financing in healthcare systems. The TEC, which is also obtained from WDI database, offers a new technical solution to healthcare systems. Moreover, government could mitigate the information asymmetry in the health sector, for instance, between patients and doctors. Government effectiveness (GE) compiled by the world government index (WGI) database in world bank captures perceptions of the quality of public services [20]. As a proxy of structural weakness, compulsory financing arrangements (SW) reflects inadequate financing and resource misallocation in healthcare systems. Thus, this paper employs compulsory financing arrangements to express structural weakness.

The rank of OPE could be employed to amplify the type of healthcare system in countries. It is widely accepted that health financing is heavily associated with the effectiveness of healthcare systems. Indeed, the high share of OPE means higher financial risk protection for households, this is also crucial for assessing the effectiveness of the healthcare system in countries [26]. If the OPE is a high percentage of total health expenditure, this would generally suggest limited financial protection, as a result of a lower effectiveness of healthcare system. Consequently, households with a higher share of OPE could contribute a higher share of their income in accessing healthcare services. Therefore, the share of OPE could be regarded as an effective tool to measure the type of healthcare system within countries. For analysis, out-of-pocket health spending has been grouped to form an inter-sectional variable. Specifically, this paper firstly computes the average value (AV) of OPE in countries over the sample period. Secondly, we sort these AV from 1 to 34 in descending order (These results are available upon request). Of those average value of OPE and the balance between different samples, this paper regards Rank 1–11 as a high-type system (H), Rank 12–22 as a middle-type system (M) and others as a low-type system (L). The details are shown in Table 2, with countries listed in groups and their abbreviation.

### 3.3. The Business Cycle Effects on Global Health Financing

**Figure 3:**
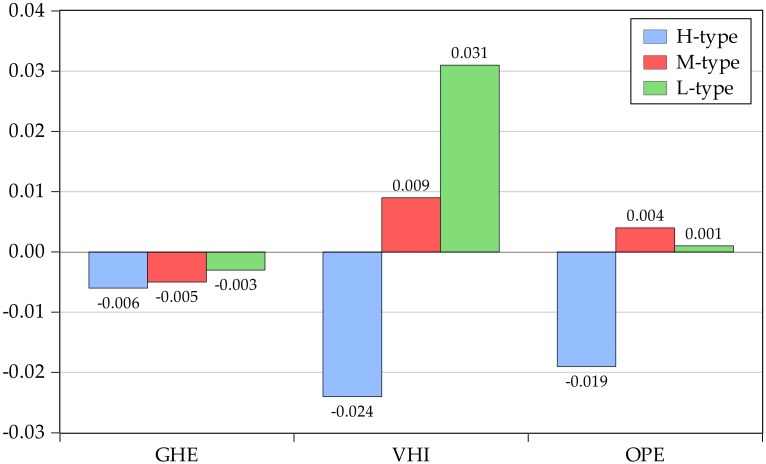
The effect of the business cycle on different types of health financing.

The quantitative investigation results are reported in Table 3 and Figure 3. Specifically, Table 3 displays the estimation of parameters in equation (4). Figure 3 snapshots the $\varphi_{10}\;$, $\varphi_{10}+\varphi_{11}$ and $\varphi_{10}+\varphi_{12}$ among the GHE, VHI, and OPE.

A comparison study of information in Table 3 reveals heterogeneous effects of the business cycle on global health financing among subsidized, voluntary and out-of-pocket health spending. More specifically, it is particularly interesting to note that there are negative effects of the business cycle on subsidized health spending. On the one hand, the inverse effect of the business cycle may be due to diseases control and social stabilization. Indeed, the downward pressure of economy in a country becomes more precarious, and the investments are uncertain as a result of the expansion of economic burden [41]. Consequently, the negative effect could increase the cost of diseases and weaken disease control, which further intensify the inequality in healthcare systems. According to the primary aim of subsidized health spending, governments or authorities began increasing the expenditure for households to access health services. Additionally, healthcare systems play crucial roles in social stabilization since their roles in basic demand for households. People may be more fearful about increasing their economic burden since the levels of living and working have been substantially weakened. It is estimated that healthcare systems could effectively improve happiness even though there are limitations to investment. On the other hand, the negative effects are also related to the quality of health. For instance, business cycles are often negatively accompanied by the spread of communicable diseases, which offer challenges to the stabilization of healthcare systems. Therefore, the business cycle has a negative effect on subsidized health spending.

As expected, the effect of the business cycle on voluntary and out-of-pocket health spending is positive. From the perspective of voluntary health spending, the fact that the positive effect of the business cycle can be fully attributed to the households’ willingness to prepay for healthcare services. For instance, proper working conditions expand the ATTI since the ATTI offers a chance to depict cash and non-cash safety. Further, the ATTI promotes the households’ willingness and consequent ascension of voluntary health spending. In terms of out-of-pocket health spending, the high dependence between economic burden and participants’ income and health conditions as a major mechanism is along expected lines. There are strong reasons to believe the economic burden effectively leads households to scale back an “affluent” lifestyle, since they reduce their expenditure on eating outside the home, and they choose to walk instead of driving. What is more, with the downward pressure of the business cycle, households rely more on private expenditure and even sell their assets to access healthcare services. As a result, they are more dependent on out-of-pocket health spending. These results indicate the recent evidence of the positive relationship between the business cycle and private health spending.

However, the picture changes a little in countries with a high-type healthcare system (H). As shown in Table 3, it is clear to note that the business cycle in these countries shows a negative effect on subsidized, voluntary and out-of-pocket health spending. These unreasonable results may reveal the role of fluctuations in the business cycle and participants’ expectations. Economic parameters such as the GDP (current US$; unit/trillion) and its volatility behave quite different in countries with diverse types of systems. Commonly, it is worth to note that the volatility of the business cycle during sample periods of High-type is higher than that in countries with a Middle- and Low-type system, which is 0.033, 0.027 and 0.028 (Source: author estimation). The intense fluctuation in countries with high-type healthcare system may weaken expectations of the household. Consequently, households spend less disposable income in the “affluent” lifestyle as a result of the deterioration of voluntary and out-of-pocket health spending. Furthermore, this paper examines to what extent the effects of the business cycle on subsidized, voluntary and out-of-pocket differ across different types of healthcare systems. The results are shown in Figure 3.

The business cycle effects on global health financing are closely related to the healthcare systems within the countries. As shown in Figure 3, it is worth to note that the impact of the business cycle in high-type countries is generally higher than that in low-type countries. This is no surprise but due to the heterogeneous effectiveness of healthcare systems. The effectiveness is heavily related to equality to access healthcare services. Indeed, high-type healthcare systems are financed through some crucial resources such as social health insurance, private health insurance, taxation and out-of-pocket payments. However, effective programs of the healthcare system require stable financing which guarantees a balance between the burden of paying according to the ATTI and benefits from health spending according to demand. Additionally, the lower effectiveness in countries with high-type systems is, the higher the financial barriers to access health services. In this vein, the business cycle in these countries more sensitively affects health financing as the limited source and less effective of healthcare systems.

## 4. The Mediating Effects on Business Cycle-Health Financing Mechanisms

This section describes empirical regularities regarding the mediating effect of external conditions and innovative financing on business-cycle-health-financing mechanism based on structural equation modeling (SEM). We first briefly develop a research design that will guide our empirical analysis to follow the procedure of SEM. Later, we introduce the mediating effect of external conditions on the business-cycle-health-financing mechanism. Finally, we also discuss the effect of business cycle on health financing through the innovative financing channel.

### 4.1. Research Design

#### 4.1.1. Methods Selected

The structural equation modeling (SEM) in this study successfully depicts the relationship among business cycle (BCycle), external conditions (ECondition), innovative health financing (IFinancing) and health financing (HFinancing) based on AMOS.24. Xiong et al. [54] offer a comprehensive review of SEM applications from perspectives of SEM design, SEM development and beneficial issues for further research based on the SEM. Since the seminal study by Bentlers [55], who models the attitude-behavior relations in psychological science based on SEM, the SEM has been employed to analyze safety behaviors, project management and urban environments, and it becomes a quasi-routine technology in the social science [56]. However, studies that measure the mediating effect of the ECondition and IFinancing using the SEM are scarce. What is more, the possibility of modeling the dependencies and testing the ambiguous form of BCycle, ECondition, IFinancing and HFinancing could be regarded as the main reason for using the SEM. On the one hand, it is difficult to construct the form of BCycle, ECondition, IFinancing and HFinancing since a single variable cannot comprehensively represent their abstract characters. On the other hand, to achieve accurate research, we also need to assess the causal relationship between BCycle, ECondition, IFinancing and HFinancing. Indeed, the SEM could be considered as a unique technology that simultaneously estimates the factor analysis and the path analysis. Compared with traditional techniques such as multivariate regression, in other words, the SEM can simultaneously explore the assessment of BCycle, ECondition, IFinancing and HFinancing as well as relationships among them [57]. Next, we briefly introduce the methodological procedure in this paper.

#### 4.1.2. Model Conceptualization

Model conceptualization, according to Table 4, focuses on constructing the measured model between BCycle, ECondition, IFinancing, HFinancing and observed variables. Specifically, observed variables are selected from theoretical reviews for background variables. For the observed variables of BCycle, large investigations indicate that real GDP, real GDP growth, nominal GDP and nominal GDP growth impact the business cycle [4,35]. As noted in Section 3.1, the real and nominal GDP respectively depicts economic conditions with(out) the impact of general price levels. Additionally, GDP and GDP growth are used to calculate the flux shape and its changes. All GDP measurements were detrended using HP-filter with a smooth parameter of 100, which could be regarded as an effective parameter to snapshot the characteristics of GDP.

In terms of ECondition, existing literature considered several main streams of potential factors, including public health conditions and economic fundamentals. It is widely accepted that Under Five Mortality and people using safely managed sanitation services contribute to public health conditions within a country and further reflect the external conditions. Other factors such as unemployment and compulsory financing arrangements of current health expenditures could be regarded as some of the external conditions. For the observed variables of IFinancing, this paper selected them based on the need to balance between inputs and outputs. Besides the patent selected in Section 3.1, we also employed gross domestic expenditure on R&D (GERD) for medical and health science (IF2) obtained from United Nations Educational, Scientific and Cultural Organization (UNESCO) database to capture the innovative input for healthcare systems. The IF2 provides additional funding for healthcare systems and also indicates the potential to expand fiscal space. Above all, GERD medical and health science is found as the input, and the number of patents is the output. They further indicate the source of IFinancing. From the perspective of HFinancing, subsidized, voluntary and out-of-pocket health spending is crucial financing into healthcare system planning. Thus, this paper selected general government expenditure on health of total general government expenditure, private prepaid plans of total expenditure on health and out-of-pocket expenditure of total expenditure on health as good measures of HFinancing.

#### 4.1.3. Path Diagram Construction

**Figure 4:**
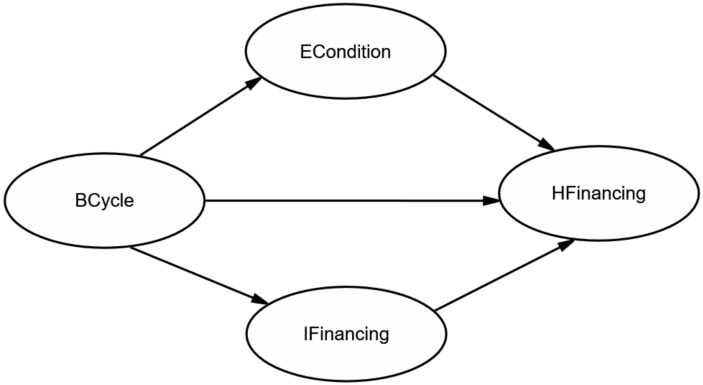
The initial structural model for global health financing.

According to the hypothesis, this paper constructed an initial structural model as shown in Figure 4. As we all know, the variables in circles were defined as latent variables. The ECondition and IFinancing are deemed as crucial paths for effectively understanding, foreseeing and achieving the goal of UHC under the control of health financing as previously mentioned in Hypotheses 2 and 3. As shown in Figure 4, “ECondition”, “IFinancing” and “HFinancing” can be thought of as endogenous variables, whereas “BCycle” is an exogenous variable.

#### 4.1.4. Model Specification and Identification

Model specification mainly focused on the goodness-of-fit measured by $\chi^{2}$ for specific SEMs. Therefore, we first estimated the SEM with all variables. Then, at least, we selected the most important observed variable in the measured model through factor analysis, which is output in SEM. We reconstructed a new SEM (Model 1) with these variables. This paper finally added variables into Model 1 and further compared $\chi^{2}$ with different SEMs. In this vein, this study could further identify the final structural model. Considering the number of observations, we separately estimated the mediating effect of external conditions and innovative financing.

#### 4.1.5. Parameter Estimation

Commonly, AMOS offers five methods of estimating discrepancy including Maximum Likelihood (ML), Generalized Least Squares (GLS), Unweighted Least Squares (ULS), Scale-free Least Squares (SFLS) and Asymptotically Distribution-Free (ADF). This paper employed the ML to estimate the final structural models and set bootstrap equal to 1000.

#### 4.1.6. Assessment of Model Fit

Table 5 represents the recommended fitness and thus considered feasibility of the analysis. In this paper, SEM models were separately estimated for the three types of countries. As displayed, the final model on the path analysis for health financing is appropriately supported. Specifically, the $\chi^{2}$, giving the value of 2.781, 5.643, 2.702, 0.911, 3.835 and 0.842 for each model, respectively, indicated acceptable fit to data. Of other parameters for goodness-of-fit, the RMSEA were below the recommended cut-off level of 0.1. Additionally, comparative fit index (CFI) for each model’s values above 0.9 offered sufficient evidence that the fit between factor analysis and data was also acceptable. In this context, this paper reports the mediating effects of external conditions in Section 4.2. The mediating effects of innovative health financing are presented in Section 4.3.

### 4.2. The Mediating Effect of External Conditions

**Figure 5:**
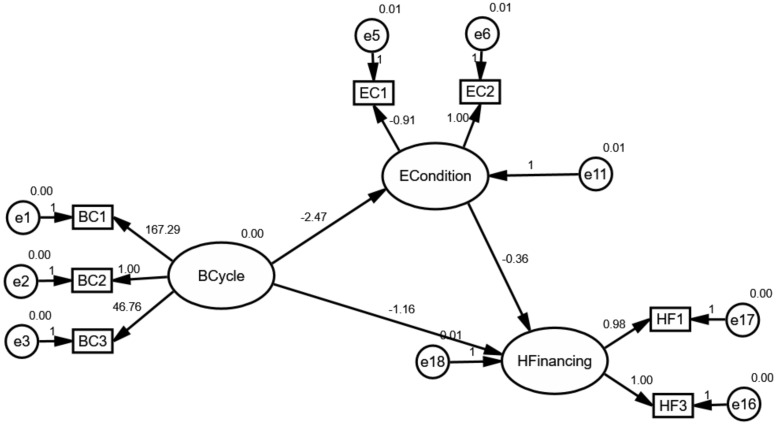
The mediating effect of external conditions in countries with high-type systems.

**Figure 6:**
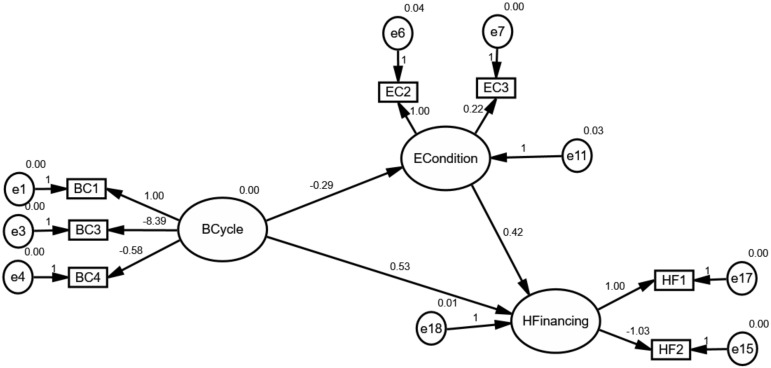
The mediating effect of external conditions in countries with middle-type systems.

**Figure 7:**
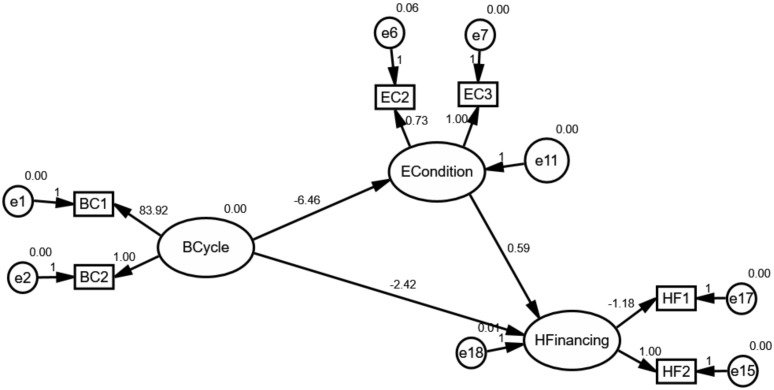
The mediating effect of external conditions in countries with the low-type system.

Figure 5, Figure 6 and Figure 7 depicts the mediating effect of external conditions in countries with high, middle and low type of the healthcare system, respectively. As seen, there were negative effects of external conditions on the relationships between the business cycles and health financing. Specifically, the results suggest that the business cycle had a strong negative effect on external conditions and as a result the increase of health financing. Besides this effect, it is evident that the business-cycle-health-financing mechanism in high-type countries depicted a different picture. Above all, the analysis elucidates that the business cycle hurt health financing through external condition channels. This finding is in line with the hypothesis. Therefore, the mediating effect of economic conditions makes it a noteworthy variable.

The results derived from the analysis clarify our theoretical hypothesis on the business-cycle-health-financing mechanism. It was found that the mediating effect of external conditions differed according to the type of healthcare system. Due to this mediating effect, it became obvious that external conditions need to be considered while adjusting the strategy of healthcare systems. External conditions are considered an important mediating factor because of two reasons: their correlation with the fiscal position of countries and the household’s demand. In terms of the fiscal position, it includes fiscal risks and financing constraints. As we all know, it could be found as expected external conditions if one country timely and professionally exposes the economic uncertainty not only in the overall economy but also in specific industry [46]. Additionally, economic diversity has displayed greater fiscal positions [6]. These relationships may reduce the fiscal risks and financing constraints, and as a consequence raise the fiscal position of countries. Thus, the business cycle plays a negative role in external conditions. However, these events could also affect the balance by altering the demand for health and healthcare service because of the stability of economic parameters according to the measurement model. Specifically, it is obviously noting that external conditions could be measured by UNF, SER and UEM. The lower values of these parameters snapshot a more stable investment environment. As a consequence, global health financing has substantially changed. From the perspective of the household’s demand, it is well understood that the business cycle exposed their willingness and changed their expectations, and eventually resulted in changes in health financing. Specifically, the main consequence of the business cycle is that it dramatically changes employment as the changes in competition for jobs [5]. This relationship expands the willingness and expectation for households to contribute the investment in other areas. Accordingly, all these agreements highlight the importance of the mediating effect of external conditions.

Moreover, the unreasonable pictures in high-type countries reveal that the mediating effect is heavily related to fluctuations in the business cycle and participants’ expectations. As mentioned in 3.2, the business cycle in countries with high-type systems has experienced more drastic volatility from 2000 to 2016 because of the ever-increasing role of sustainable development all over the world compared with other groups. This volatility may be regarded as an important source, affecting the uncertainty of external conditions and households’ expectations. Consequently, the business cycle in high-type countries plays a negative effect on external conditions. Furthermore, this effect could trigger the uncertainty of health spending since households could expect to find higher wages to satisfy the normal and unexpected expenditure for healthcare service when facing an uncertain environment. What is more, the intense volatility may also weaken expectations of households. Consequently, even economic parameters are better, households spend less disposable income in the “affluent” lifestyle or investment.

### 4.3. The Mediating Effect of Innovative Health Financing

**Figure 8:**
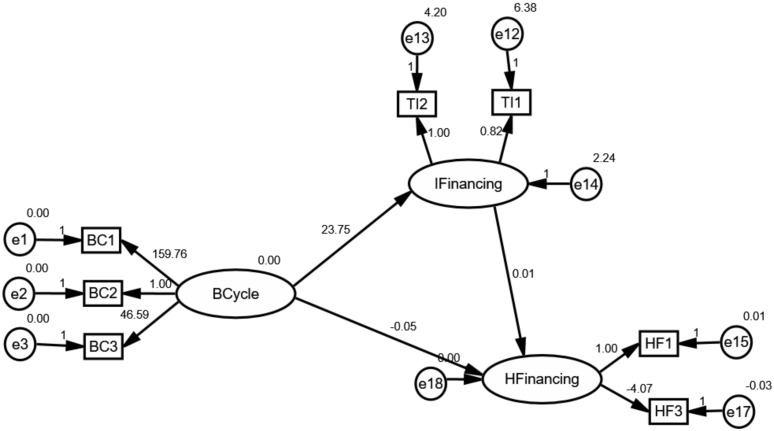
The mediating effect of innovative health financing in countries with high-type systems.

**Figure 9:**
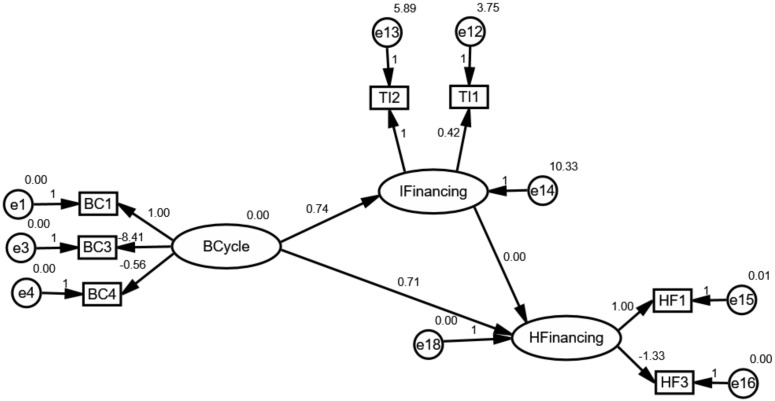
The mediating effect of innovative health financing in countries with middle-type systems.

**Figure 10:**
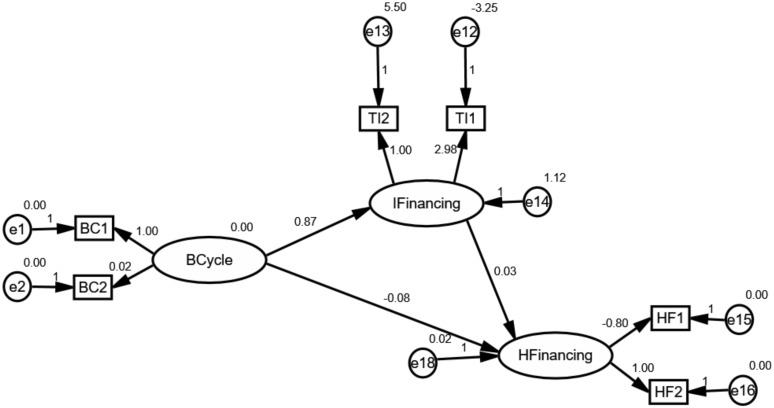
The mediating effect of innovative health financing in countries with low-type systems.

Some interesting results, associated with the mediating effect of IFinancing, are shown in Figure 8, Figure 9 and Figure 10. As seen, IFinancing may positively mediate the effect of the business cycle on health financing. Specifically, the results reveal that innovative financing is a required mediating variable between the business cycle and health financing. Besides, it is worth to note that the mediating effect could be associated with types of the healthcare system. In opposition to external conditions, the analysis elucidates that the business cycle positively affects global health financing through innovative health financing channels. Although innovative health financing does not behave to be mediating in affecting health financing in middle-type countries, its correlation, as well as the heterogeneous effect on health financing, makes it a noteworthy variable.

The findings derived from the analysis are in line with our hypothesis. We observed that IFinancing had a positive mediating effect on the correlation between BCycle and HFinancing. We see that effective control of innovative health financing would ultimately handle better financing for the healthcare system in high- and low-countries. That innovative health financing was found to be of positive effect reveals the crucial role of guarantee of sustainable financing and resource allocation. Indeed, the business cycle offered the uncertainty/opportunity of economic equality which would create effectiveness for transferring traditional funds, further resulting in a major debate on the sustainability of financing. Consequently, this relationship changes global health financing, since the effectiveness of transferring could attract more innovative health financing. For another, the business cycle also showed its effect on health financing and assisting in resource allocation. The business cycle also changed the conversion efficiency in healthcare systems as a result of the slowdown in the competitiveness of the economy and the consequent change in share of resource allocation. As a result, this change may create uncertainty in accessing healthcare services. What is more, inequalities associated with regional and social resource allocation left the poor with a greater unmet demand for healthcare services. Accordingly, all these agreements highlight the positive effect of the business cycle on health financing through innovative health financing channels.

Moreover, the diverse results in countries with different types of healthcare systems reveal that the mediating effect heavily is related to sharing equally, political environment and the effectiveness of innovative protection. On the one hand, although there are positive mediating effects of innovative health financing on the relationship between the business cycle and global health financing, these gains have not been shared equally because of the diverse access for healthcare service [50]. In this sense, health financing is not affected by innovative health financing in middle-type countries as shown in Figure 9. The BCycle increases the IFinancing, while the IFinancing does not affect HFinancing. On the other hand, political stability such as the trajectory of flat financing may continue and will increase the reliance on domestic and innovative financing sources to sustain and scale health programs in low-type countries. Furthermore, this uncertainty could change sustainable financing. What is more, these also require that innovative financing has effectively pooled and channeled financing from innovative health financing to beneficiary countries. For instance, innovative instruments, such as exchange-traded funds failed to generate any valuable financing. Airline Levy, however, has become a steady source of global health financing [49]. Since the innovative financing appears to positively mediate the business-cycle-health-financing mechanism, therefore, managers should improve the effectiveness between the R&D expenditures and the actual use in healthcare services.

## 5. Conclusions

The anticipation of global health financing with regards to the business cycle is vital for promoting UHC. The empirical results with a hierarchical linear model and structural equation modeling all point to the heterogeneous effect of the business cycle on health financing from 2000 to 2016, disaggregated by the source of financing. In this paper, we first explore the effect of the business cycle on global health financing among subsidized, voluntary and out-of-pocket health spending based on a hierarchical linear model. Furthermore, this paper investigates the mediating effect of external conditions and innovative health financing with a framework of structural equation modeling. Specific conclusions are as follows.

The business cycle effects in healthcare systems have stronger differences among sources of health financing. In general, the business cycle has a negative impact on subsidized spending, whereas the effect of the business cycle on voluntary and out-of-pocket health spending is positive. According to the differences in the fluctuations in the business cycle and participants’ expectations among different countries, we found that business-cycle-health-financing mechanism in countries with high-type healthcare system is fundamentally different from that in other countries. This result is further supported by the fact cyclical fluctuation is now a common event rather than rare occurrence. For one thing, health financing could be regarded as a tool to determine how pressures on healthcare system are weathered without loss of equity, quality and protection. Moreover, out-of-pocket health spending is not acceptable on sustainable healthcare grounds. For another, this conclusion indicates competitiveness gains which include the changes of subsidized health spending are effective ways for the economy to adjust during different phases of the business cycle.

The external conditions negatively mediate the relationship between the business cycle and health financing in general. Relative changes in external conditions with health financing can reflect its mediating effect in the mechanism. However, we also find that an external condition also exerts its positive mediating effect on business cycle-health financing mechanisms. These results further indicate the important role of expectations of households. A better organization of external conditions from official channels, reinforcing the informative share between household within healthcare services, could help regulate their expectation, thus, improve the equity to access healthcare system.

The business cycle effects have been of a beneficial shock on global health financing through the innovative health financing channel, but it is heterogeneous in countries with different types of healthcare systems. At a more specific level, we conclude that the mediating effect of innovative health financing will be mitigated when in middle-type countries. Additionally, innovative health financing shows a positive effect on the business cycle, as well as health financing in countries with both high- and low-type of healthcare systems. As innovative financings, both patents and R&D expenditure in medical service, increase, and public health services are pushed to higher, it is evident that healthcare systems through innovative financing can be more responsive to economic conditions and more effective in health spending consolidation.

These findings have implications to households and policymakers in healthcare systems. For households, the primary aim is to reduce their private spending in healthcare systems. They have a better focus on the health policy and are familiar with the market and economic conditions because of the asymmetry of information. In terms of policymakers, particularly in middle-income countries, they need to increase their effort to shift innovative health financing towards actual use if the goal of UHC is to be realized. Additionally, the fluctuations in the business cycle and participants’ expectations play a deterministic role on the mediating effect. Thus, policymakers also need to build equality to access the healthcare service and guide a reasonable expectation, such as setting up a special R&D expenditure, strengthening the supervision of innovative findings and their industrialization and improving the sustainable development of healthcare service.

This study bears several limitations. Despite these results presented in the HLM and SEM analysis, this paper neglects the heterogeneous effect of the business cycle during different phases, as well as the diverse fundamental of economy and complex expectations of market participants during economic recessions or expansion [58]. Thus, we could further explore this effect by dividing the sample periods into economic recessions and expansions. Moreover, further studies about the moderating effect of external conditions, innovative health financing and other factors among different income countries would be a valuable area to investigate. What is more, a new analysis of relationships between business or credit cycle and financing schemes could be regarded as a valuable area.

## Figures and Tables

**Table 1:** Variables selection for the hierarchical linear model (HLM).

Nature of Variables	Variables	Abbr.	Measurement	Description	Source
Dependent variables	Government Health Expenditure	GHE	Domestic General Government Health Expenditure as % Current Health Expenditure	This indicator depicts that governments give subsidy to healthcare systems.	GHE database
	private prepaid plans	VHI	Voluntary Financing Arrangements as % of Current Health Expenditure	This shows the voluntary prepayment schemes to healthcare systems.	GHE database
	Out-of-Pocket Expenditure	OPE	Out-of-pocket as % of Current Health Expenditure	This highlights the importance of assessing the extent of financial protection in healthcare systems.	GHE database
Explanatory variable	Business Cycle	BC	Real GDP	This variable reflects the economic operation within a country.	IFS database
Control variables	Under Five Mortality rate	UNF	Mortality rate, under-5 (per 1,000 live births)	This indicator concerns about the global monitoring of child mortality.	WDI database
	People using safely managed sanitation services	SER	People using safely managed sanitation services (% of population)	This indicator indicates the percentage of people using improved sanitation facilities that are not shared with other households and where excreta are safely disposed of in situation or transported and treated offsite.	WDI database
	Government Effectiveness	GOV	Government Effectiveness	This indicator represents a proxy for the quality of government.	WGI database
	Unemployment	UEM	Unemployment, total (% of total labor force)	This indicator is of critical importance in measuring the (in)ability of workers to readily obtain gainful work within a country.	WDI database
	Patent applications	TEC	Patent applications, residents	Patent applications are worldwide patent applications filed through the Patent Cooperation Treaty procedure or with a national patent office for exclusive rights for an invention-a product or process that provides a new way of doing something or offers a new technical solution to a problem.	WDI database
	Compulsory Financing Arrangements	SW	Compulsory Financing Arrangements as % of Current Health Expenditure	This reflects inadequate financing and resource misallocation in healthcare systems.	GHE database ^1^

^1^ Note: 1. Abbr. means the “Abbreviation”. 2. GHE database represents the “Global Health Expenditure database”. IFS database means the “IMF (International Monetary Fund) International Financial Statistics database”. WDI database is the “World Development Indicators database”. WGI database represents the “World Governance Indicators database”.

**Table 2:** The results of the sample divided.

Categories	Rank	Abbr.	Country Lists
High OPE	1–11	H	Armenia, Azerbaijan, Bangladesh, Ecuador, Egypt, Georgia, Guatemala, Morocco, Iran, Pakistan, Philippines
Middle OPE	12–22	M	Bosnia and Herzegovina, China, Korea, Malaysia, Mexico, Paraguay, Peru, Singapore, Tunisia, Uzbekistan, Venezuela
Low OPE	23–34	L	Algeria, Belarus, Brazil, Jordan, Kazakhstan, Mongolia, New Zealand, Norway, Russian Federation, Saudi Arabia, United Kingdom, United States ^2^

^2^ Note: Abbr. means the “Abbreviation”.

**Table 3:** The estimation of equation (4).

HSP	Parameters	Coef.	St. D	t-Value	Prob.
GHE	$\;\varphi_{00}\;$	0.661	0.029	23.14	0.000
	$\;\varphi_{01}\;$	−0.362	0.041	−8.766	0.000
	$\;\varphi_{02}\;$	−0.169	0.041	−4.116	0.000
	$\;\varphi_{10}\;$	−0.003	0.017	−0.149	0.882
	$\;\varphi_{11}\;$	−0.003	0.212	−0.159	0.874
	$\;\varphi_{12}\;$	−0.002	0.024	−0.093	0.927
	C.V.	YES			
	Sum.	H		−0.006	
		M		−0.005	
		L		−0.003	
VHI	$\;\varphi_{00}\;$	0.333	0.028	12.070	0.000
	$\;\varphi_{01}\;$	0.351	0.039	8.811	0.000
	$\;\varphi_{02}\;$	0.175	0.039	4.391	0.000
	$\;\varphi_{10}\;$	0.031	0.020	1.504	0.133
	$\;\varphi_{11}\;$	−0.055	0.024	−2.246	0.025
	$\;\varphi_{12}\;$	−0.022	0.027	−0.804	0.422
	C.V	YES			
	Sum.	H		−0.024	
		M		0.009	
		L		0.031	
OPE	$\;\varphi_{00}\;$	0.237	0.023	10.353	0.000
	$\;\varphi_{01}\;$	0.388	0.033	11.716	0.000
	$\;\varphi_{02}\;$	0.182	0.033	5.500	0.000
	$\;\varphi_{10}\;$	0.001	0.077	0.016	0.987
	$\;\varphi_{11}\;$	−0.020	0.094	−0.211	0.833
	$\;\varphi_{12}\;$	0.003	0.105	0.028	0.978
	C.V.	YES			
	Sum.	H		−0.019	
		M		0.004	
		L		0.001 ^3^	

^3^ Note: 1. Coef. stands for the coefficient. St. D represents the “Standard error”. Prob. is the probability of estimation and sum. means the summary of the effects of the business cycle among countries with different types of healthcare system. 2. C.V. presents the “Control variables” and YES stands model estimation includes control variables. The backward elimination is employed to select control variables because of their significance among different health financing. In this vein, control variables include UNF, GOV, TEC, and SW when HSP is GHE. When VHI is regarded as the HSP, control variables are UNF, SER, GOV, UEM, TEC, and SW. However, control variables are SER, GOV, and SW in the OPE. The full results of equation (4) are shown in Table A1.

**Table 4:** Variables selection for structural equation modeling (SEM).

Latent Variables	Observed Variables	Abbr.	Source
Business cycle (BCycle)	Real GDP	BC1	IFS database
	Real GDP growth	BC2	IFS database
	Nominal GDP	BC3	IFS database
	Nominal GDP growth	BC4	IFS database
External conditions (ECondition)	Under five mortality rate	EC1	WDI database
	People using safely managed sanitation services	EC2	WDI database
	Unemployment	EC3	WDI database
	Compulsory Financing Arrangements of Current Health Expenditure	EC4	GHE database
Innovative financing (IFinancing)	Number of patents	IF1	WDI database
	GERD Medical and health science	IF2	UNESCO database
Health financing (HFinancing)	General government expenditure on health of total general government expenditure	HF1	GHE database
	Private prepaid plans of total expenditure on health	HF2	GHE database
	Out-of-pocket expenditure of total expenditure on health	HF3	GHE database

**Table 5:** Assessment of model fit.

Model	$\chi^{2}$	Prob.	RMSEA (0 to 0.1)	ECVI	NCP	PGFI (>0.5)	CFI (>0.9)
H1	2.781	0.993	0.000	0.198 [0.242, 0.242]	0.000 [0, 0.000]	0.391	1.000
H2	5.643	0.896	0.000	0.213 [0.242, 0.255]	0.000 [0, 2.405]	0.390	1.000
M1	2.702	0.994	0.000	0.197 [0.242, 0.242]	0.000 [0, 0.000]	0.391	1.000
M2	0.911	1.000	0.026	0.188 [0.242, 0.242]	0.000 [0, 0.000]	0.392	1.000
L1	0.795	0.992	0.000	0.167 [0.177, 0.177]	0.000 [0, 0.000]	0.284	1.000
L2	0.842	0.991	0.000	0.152 [0.177, 0.177]	0.000 [0, 0.000]	0.285	1.000 ^5^

^5^ Note: 1. Model H1 stands for the exploration of mediating effects of external conditions in countries with high-type systems, and H2 represents the mediating effect of innovative health financing. Similarly, Model M1 and M2 separately depict the mediating role of external conditions and innovative health financing towards the relationship between business cycle and health financing in medium-type countries. Model L1 explores the effect of business cycle on health financing through external conditions channel in low-type countries, whereas Model L2 stands for the mediating effect of innovative health financing. 2. Prob. means the “Probability of $\chi^{2}$ ”. RMSEA is the “root mean square error of approximation”. ECVI means the “expected cross-validation index”. NCP is the “non-centrality parameter”. PGFI stands for the “parsimonious goodness of fit index”, and CFI represents the “comparative fit index”. 3. The recommended levels of statistics are shown in parentheses, and the confidence level is shown in square brackets.

## Appendix A. Full Results of the HLM

**Table A1:** Full results of model (4).

HSP	Parameters	Coef.	St. D	Statistic-Value	Prob.
GHE	$\;\varphi_{00}\;$	0.661	0.029	23.14	0.000
	$\;\varphi_{01}\;$	−0.362	0.041	−8.766	0.000
	$\;\varphi_{02}\;$	−0.169	0.041	−4.116	0.000
	$\;\tau_{t,i}\;$	0.098	0.009	101,101.5	0.000
	$\;\varphi_{10}\;$	−0.003	0.017	−0.149	0.882
	$\;\varphi_{11}\;$	−0.003	0.212	−0.159	0.874
	$\;\varphi_{12}\;$	−0.002	0.024	−0.093	0.927
	$\;\pi_{2,i}\;$	0.017	0.004	4.104	0.000
	$\;\pi_{4,i}\;$	0.005	0.001	2.886	0.005
	$\;\pi_{6,i}\;$	0.001	0.000	2.333	0.020
	$\;\pi_{7,i}\;$	0.988	0.005	186.2	0.000
VHI	$\;\varphi_{00}\;$	0.333	0.028	12.070	0.000
	$\;\varphi_{01}\;$	0.351	0.039	8.811	0.000
	$\;\varphi_{02}\;$	0.175	0.039	4.391	0.000
	$\;\pi_{t,i}\;$	0.095	0.009	71,782.03	0.000
	$\;\varphi_{10}\;$	0.031	0.020	1.504	0.133
	$\;\varphi_{11}\;$	−0.055	0.024	−2.246	0.025
	$\;\varphi_{12}\;$	−0.022	0.027	−0.804	0.422
	$\;\pi_{2,i}\;$	−0.034	0.005	−6.162	0.000
	$\;\pi_{3,i}\;$	0.021	0.007	3.042	0.003
	$\;\pi_{4,i}\;$	−0.004	0.002	−2.036	0.042
	$\;\pi_{5,i}\;$	−0.001	0.001	−1.850	0.064
	$\;\pi_{6,i}\;$	0.066	0.019	3.489	0.001
	$\;\pi_{7,i}\;$	−1.021	0.006	−165.1	0.000
OPE	$\;\varphi_{00}\;$	0.237	0.023	10.353	0.000
	$\;\varphi_{01}\;$	0.388	0.033	11.716	0.000
	$\;\varphi_{02}\;$	0.182	0.033	5.500	0.000
	$\;\pi_{t,i}\;$	0.079	0.006	3306.75	0.000
	$\;\varphi_{10}\;$	0.001	0.077	0.016	0.987
	$\;\varphi_{11}\;$	−0.020	0.094	−0.211	0.833
	$\;\varphi_{12}\;$	0.003	0.105	0.028	0.978
	$\;\pi_{3,i}\;$	0.049	0.023	2.119	0.034
	$\;\pi_{4,i}\;$	−0.020	0.007	−2.597	0.010
	$\;\pi_{7,i}\;$	−0.744	0.024	−31.481	0.000
